# Quality of life in patients with non-small cell lung cancer treated with PD-1/PD-L1 inhibitors: a systematic review and meta-analysis

**DOI:** 10.1186/s12957-022-02800-1

**Published:** 2022-10-04

**Authors:** Wei Liu, Qian Zhang, Tiantian Zhang, Li Li, Chunhua Xu

**Affiliations:** 1 Department of Respiratory Medicine, Affiliated Nanjing Brain Hospital, Nanjing Medical University, Nanjing, 210029 Jiangsu China; 2 Clinical Center of Nanjing Respiratory Diseases and Imaging, Nanjing, 210029 Jiangsu China

**Keywords:** PD-1/PD-L1 inhibitors, Chemotherapy, Quality of life, Non-small cell lung cancer, Meta-analysis

## Abstract

**Background:**

Immune checkpoint inhibitors (ICIs) have dramatically prolonged survival in non-small cell lung cancer (NSCLC) patients, but little research had focused on its impact on quality of life (QoL). The purpose of our study was to compare the QoL in patients with NSCLC treated with programmed cell death protein-1/programmed cell death-ligand 1 (PD-1/PD-L1) inhibitors versus chemotherapy.

**Methods:**

We searched for randomized controlled trials utilizing the Quality of Life Questionnaire Core 30 items (QLQ-C30) and the EuroQol Five Dimensions Questionnaire-3L (EQ-5D-3L) to assess the QoL of NSCLC treated with PD-1/PD-L1 inhibitors versus chemotherapy. We collected hazard ratios (HRs) for the time from baseline to the first clinically significant deterioration (TTD) and effect size as the difference in mean change between and within treatment groups in patients’ reported outcomes (PROs). (PROSPERO registration number: CRD42022296680).

**Results:**

In the five trials reported by QLQ-C30, TTD was longer in PD-1/PD-L1 inhibitors compared with control groups (*HR* = 0.86; 95% *CI* = 0.76, 0.97; *P* = 0.013), with similar results in terms of physical function, role function, and pain. The difference in mean change between the PD-1/PD-L1 inhibitors group and the chemotherapy group in QLQ-C30 and EQ-5D VAS was 3.64 (95% *CI* = 1.62, 5.66; *P* = 0.001) and 4.74 (95% *CI* = 2.65, 6.83; *P* = 0.001), which supported PD-1/PD-L1 inhibitors. However, for the EQ-5D utility index, there was no statistically significant difference between the two groups, with a mean change difference of 0.03 (95% *CI* = −0.01, 0.07; *P* = 0.094). The mean change from baseline to follow-up in PD-1/PD-L1 inhibitors group was 2.57 (95% *CI* = 0.43, 4.71; *P* = 0.019), and chemotherapy group was −1.31 (95% *CI* = −3.71, 1.09; *P* = 0.284), correspondingly. The subgroup analysis showed that no difference was observed between open-label and double-blind trials in patients treated with chemotherapy or PD-1/PD-L1 inhibitors.

**Conclusion:**

In conclusion, PD-1/PD-L1 inhibitors could improve the QoL of patients with NSCLC compared to chemotherapy and reduce unfavorable symptoms during treatment.

## Background

ICIs work by regulating T-cell cytotoxicity to tumors as well as activating cells of the innate and adaptive arms, mainly including cytotoxic T-lymphocyte-associated antigen 4 (CTLA-4), PD-1, and PD-L1 [1]. NSCLC accounts for approximately 85% of lung cancers, and PD-1/PD-L1 inhibitors alone or combined with chemotherapy are the first-line palliative treatment for unresectable NSCLC and have been incorporated into neoadjuvant therapy for resectable patients with NSCLC [2, 3]. The two phase 3 trials (CheckMate 017 and CheckMate 057) reported that 5-year PFS rates and OS rates of nivolumab-treated patients and docetaxel-treated patients in previously treated advanced NSCLC were 8.0% versus 0% and 13.4% versus 2.6%, respectively, and nivolumab showed the superiority survival benefit than docetaxel [4]. However, ICIs can also lead to immune-related adverse events by releasing autoreactive T cells, such as immune-related pneumonia, vomiting, rash, and diarrhea [5].

QoL is a subjective scale to collect the severity of patients’ symptoms at a specific time point. QoL not only is an independent prognostic factor for lung cancer patients, but also evaluates the benefit-risk ratio of immune checkpoint inhibitors [6, 7]. One of the most common questionnaires used to evaluate patients with lung cancer is the QLQ-C30, which consists of five functional scales (physical, role, cognitive, emotional, and social) and nine symptom scales (fatigue, pain, nausea and vomiting, dyspnea, appetite loss, constipation, diarrhea, sleep disturbance), as well as a global health and quality-of-life scale [8]. The EQ-5D-3L primarily consists of the EQ-5D utility index and the EQ visual analog scale (VAS). EQ-5D-3L describes five dimensions of health (mobility, self-care, usual activities, pain/discomfort, and anxiety/depression), and three levels in each dimension represent no problems, moderate problems, and extreme problems, respectively [9, 10]. EQ-5D-3L is a preference-based measure that generates an index-based summary score. The EQ-5D utility index score is typically interpreted along a continuum where 1 represents the best possible health and 0 represents dead [11, 12]. Few studies have systematically assessed the QoL of ICIs in patients with NSCLC due to the heterogeneity in the quality assessment of phases 2 to 3 clinical trials in patients with various types of cancer. A series of 10 eligible trials were included in this meta-analysis, and the QLQ-C30 and EQ-5D-3L questionnaires were applied to assess the QoL of patients with NSCLC in terms of TTD and the differences in mean change between and within groups. The study was designed to systematically investigate whether PD-1/PD-L1 inhibitors (atezolizumab, avelumab, durvalumab, nivolumab, pembrolizumab) improved the QoL of patients with NSCLC compared to chemotherapy based on published randomized controlled trials.

## Materials and methods

### Search strategy and study selection

Study selection corresponded with the Preferred Reporting Items for Systematic Reviews and Meta-Analyses statement [13]. This study was registered on PROSPERO (ID no. CRD42022296680). We searched PubMed, Embase, and the Cochrane Library databases. The search deadline was on 15 January 2022. Search terms were as follows: ((atezolizumab) OR (avelumab) OR (durvalumab) OR (nivolumab) OR (pembrolizumab)) AND ((non-small cell lung cancer) OR (carcinoma, non-small cell lung) OR (carcinomas, non-small cell lung) OR (lung carcinoma, non-small cell) OR ((non-small cell lung carcinomas) OR (non-small cell lung carcinoma) OR (lung carcinomas, non-small cell) OR (carcinoma, non-small cell lung) OR (non-small cell lung carcinoma) OR (non-small cell lung cancer)) AND ((life quality) OR (health-related quality of life) OR (health-related quality of life) OR (hrqol)).

Inclusion criteria were as below:

- Patients with NSCLC were studied.
- The trial group received PD-1/PD-L1 inhibitors excluding single CTLA-4, while the control group received standard platinum-based chemotherapy.
- PROs were reported.

**Fig. 1:**
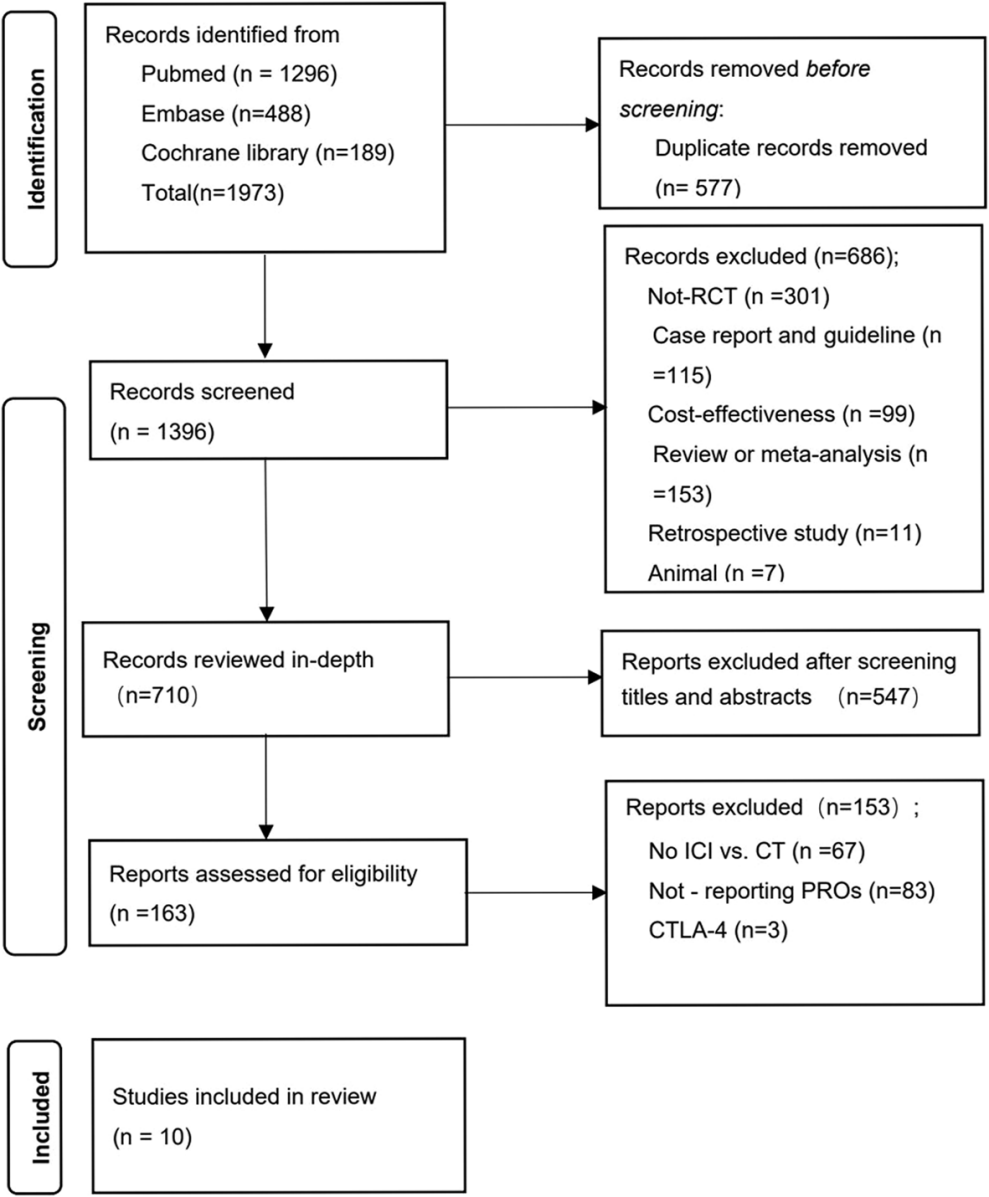
The PRISMA flowchart: the selection process for the eligible studies

We excluded reviews, case reports, and retrospective studies (Fig. 1).

### Data extraction

The extracted data includes trial name, first author, phase, masking, study type, NCT number, QoL measure, ECOG PS, follow-up, intervention arm, comparison arm, TTD, and difference in mean change between and within groups from baseline to follow-up (Table 1).

**Table 1:** Characteristics of the studies included in the meta-analysis

Trial name	First author, year	Phase	Masking	Study type	NCT number	QoL measure	ECOG PS	Treatment arms	Follow-up, week
KEYNOTE-010 [14]	Barlesi F., 2019	II/III	Open label	RCT	NCT01905657	EQ-5D-3L, EORTC QLQ-C30 EORTCQLQ-LC13	0–1	Pembrolizumab 2 mg/kg Q3W, pembrolizumab 10 mg/kg Q3W Docetaxel 75 mg/m^2^ Q3W	12
CheckMate 057 [15]	Reck M., 2018	III	Open label	RCT	NCT01673867	LCSS, EQ-5D 3L	0–1	Nivolumab 3 mg/kg Q2W Docetaxel 75 mg/m^2^ Q3W	66
KEYNOTE-024 [16]	Brahme J. R., 2017	III	Open label	RCT	NCT02142738	EORTC QLQ-C30 EORTC QLQ-LC13, EQ-5D-3L	0–1	Pembrolizumab 200 mg Q3W Five platinum-based chemotherapy regimens Q3W: carboplatin + pemetrexed (500 mg/m^2^), cisplatin (75 mg/m^2^) + pemetrexed (500 mg/m^2^), carboplatin + gemcitabine (1250 mg/m^2^), cisplatin (75 mg/m^2^) + gemcitabine (1250 mg/m^2^), or carboplatin + paclitaxel (200 mg/m^2^)	15
OAK [17]	Bordoni R., 2018	III	Open label	RCT	NCT02008227	EORTCQLQ-C30 EORTC QLQ-LC13.	0–1	Atezolizumab 1200 mg Q3W Docetaxel 75 mg/m^2^ Q3W	15
CheckMate 017 [18]	Reck M., 2017	III	Open label	RCT	NCT01642004	LCSS, EQ-5D	0–1	Nivolumab 3 mg/kg Q2W Docetaxel 75 mg/m^2^ Q3W	60
KEYNOTE-407 [19]	Mazieres J., 2018	III	Double blind	RCT	NCT02775435	EORTC QLQ-C30, EORTC QLQ-LC13, EQ-5D-3L	0–1	Pembro 200 mg Q3W Pbo Q3W + carboplatin AUC 6 and paclitaxel 200 mg/m^2^ Q3W or nab-paclitaxel 100 mg/m^2^ QW	18
PACIFIC [20]	Hui R., 2019	III	Double blind	RCT	NCT02125461	EORTC QLQ-C30 EORTC QLQ-LC13, EQ-5D	0–1	Durvalumab 10 mg/kg Q2W Matching placebo 1−42 days after chemoradiotherapy	48
EVIDENS [21]	Pero l. M., 2019	N/A	Double blind	RCT	NCT03382496	EQ-5D-3L	0–1	Nivolumab NA	36
KEYNOTE-189 [22]	Garassino M. C., 2020	III	Double blind	RCT	NCT02578680	EORTC QLQ-C30 EORTC QLQ-LC13	0–1	Pembrolizumab (200 mg) or saline placebo Q3W Pemetrexed (500 mg/m^2^) with carboplatin (5 mg/mL per min) or cisplatin (75 mg/m^2^, investigator’s choice) Q3W for four cycles, followed by pemetrexed maintenance therapy Q3W	21
MYSTIC (D/D + T) [23]	Garon E. B., 2021	III	Open label	RCT	NCT02453282	EORTC QLQ-C30 EORTC QLQ-LC13	0–1	Durvalumab 20 mg/kg Q4W, durvalumab 20 mg/kg Q4W plus tremelimumab 1 mg/kg Q4W Investigator’s choice of platinum-based doublet chemotherapy (four to six cycles)	24

### Statistical analysis

The primary outcomes of our study are as follows: (a) TTD was defined as the time from baseline to first clinically significant deterioration in PROs. A ≥ 10 points score changes for the QLQ-C30, 0.08 for the EQ-5D utility index, and 7 points for the EQ-5D VAS were deemed clinically relevant. (b) The pooled difference mean change is between and within groups from baseline to follow-up, which was all assessed by QLQ-C30 and EQ-5D-3L. We collected hazard ratios (HRs) for TTD and performed effect size as the difference in mean change in PROs between and within treatment groups. We performed a methodological quality assessment of the enrolled trials using the Cochrane risk-of-bias tool, which consists of six items: random sequence generation, allocation concealment, blinding of participants and personnel, blinding of outcome assessment, and incomplete outcome data, selective reporting, and other bias. Each risk-of-bias item was classified as high risk, low risk, and unclear risk [24]. We use the Consolidated Standards of Reporting Trials (CONSORT) PRO checklist to assess the quality of PRO [25]. Two authors (WL and QZ) independently extracted data and performed quality assessment in this process, and discrepancies were resolved by consensus (CHX). Each pooled effect size was estimated by means of the random effects model according to the DerSimonian and Laird method [26]. Heterogeneity was assessed using Cochran’s Q statistic and *I*^2^ statistic. *I*^2^ values of 25%, 50%, and 75% represented low, moderate, and high heterogeneity, respectively. Beggʼs and Eggerʼs tests were used to assess publication bias for meta-analyses for QoL. Sensitivity analyses were also conducted. *P* < 0.05 was considered statistically significant difference. All analyses were performed by using the Stata 16.0 software except for quality assessment which was performed using Review Manager 5.3.

## Results

### Literature search and characteristics of the studies

**Fig. 2:**
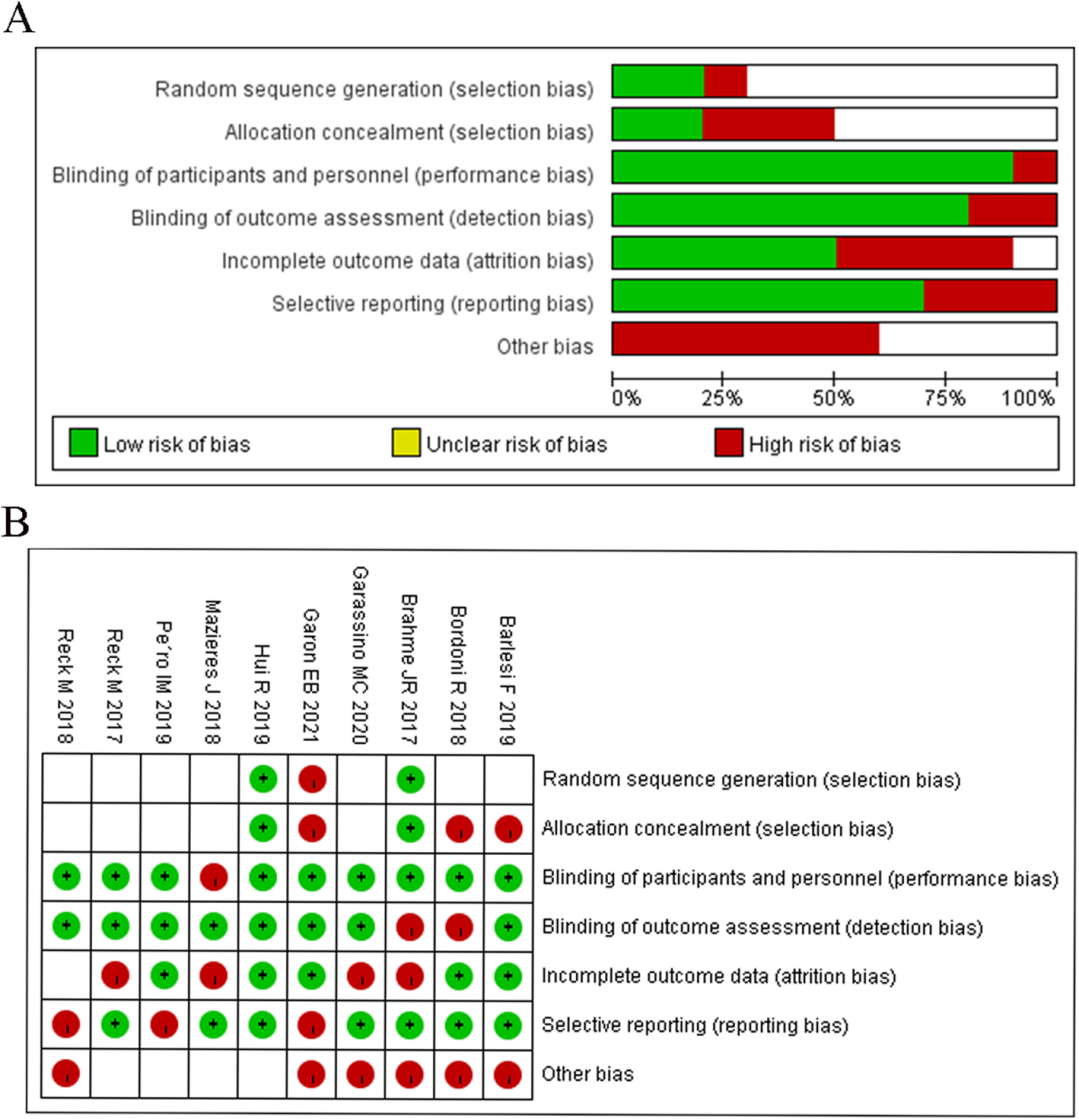
Quality assessment of the eligible studies in risk-of-bias graph (**A**) and risk-of-bias summary (**B**)

We searched the PubMed, Embase, and Cochrane Library databases for a total of 1973 publications according to the PICOS principles, of which 577 duplicates were removed, and a total of 10 publications met the inclusion criteria after screening (Fig. 1). There were eight phase 3 trials in this study; one was phase II/III, and the other was not explained in the original study; among those, clinical trials were performed with pembrolizumab (*n* = 4), nivolumab (*n* = 3), durvalumab (*n* = 2), and atezolizumab (*n* = 1). Eastern Cooperative Oncology Group Performance Status (ECOG PS) was 0-1. The follow-up period ranged from 12 to 66 weeks with a median of 22.5 weeks. PRO tools involved in these trials included EQ-5D-3L, EORTC QLQ-C30, EORTC QLQ-LC13, and LCSS, the most common of which are EORTC QLQ-C30 and EQ-5D-3L (*n* = 7, 70%) (Table 1). Quality assessment was reported in Fig. 2.

### Time from baseline to first deterioration

**Fig. 3:**
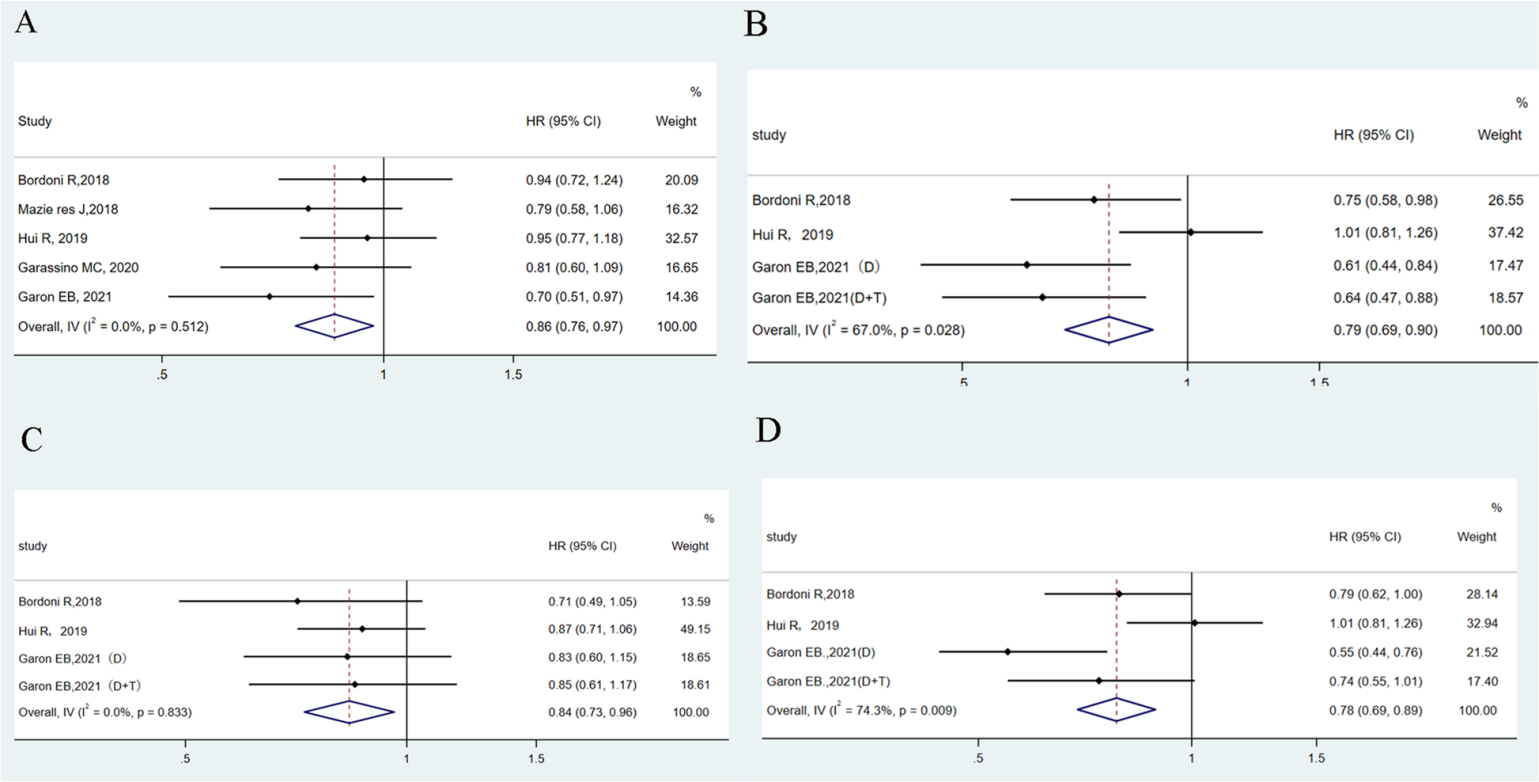
Forest plot of hazard ratios for the time from baseline to first deterioration in the Quality of Life Questionnaire Core 30 items on quality of life (**A**), physical function (**B**), pain (**C**), and role function (**D**). **A** Random effect: *P* = 0.013; Egger’s test: *P* = 0.057; Begg’s test: *P* = 0.027. **B** Random effect: *P* = 0.000; Egger’s test: *P* = 0.020; Begg’s test: *P* = 0.089. **C** Random effect: *P* = 0.000; Egger’s test: *P* = 0.226; Begg’s test: *P* = 0.308. **D** Random effect: *P* = 0.012; Egger’s test: *P* = 0.293; Begg’s test: *P* = 0.308

**Fig. 4:**
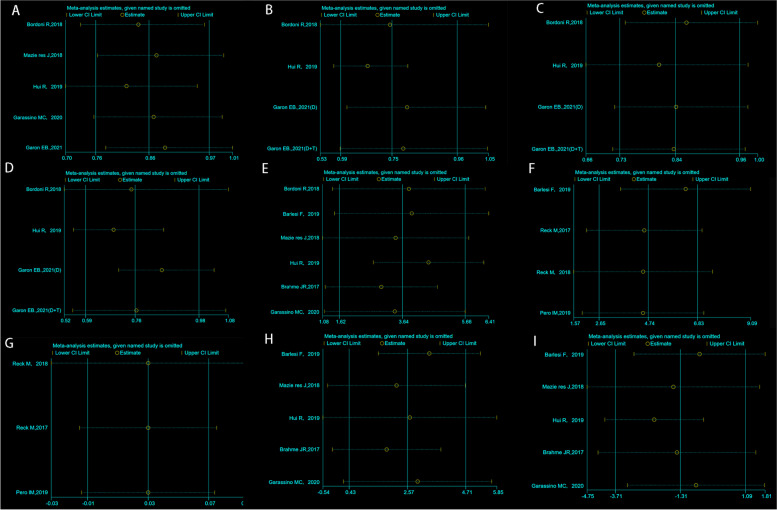
Sensitivity analyses of time from baseline to first deterioration in the Quality of Life Questionnaire Core 30 items on quality of life (**A**), physical function (**B**), pain (**C**), and role function (**D**). Sensitivity analyses of mean change from baseline to follow-up between groups for the Quality of Life Questionnaire Core 30 items (**E**), EQ-5D VAS (**F**), and EQ-5D utility index (**G**). Sensitivity analyses of difference in mean change from baseline to follow-up within groups: PD-1/PD-L1 inhibitors (**H**) and controls (**I**)

A total of 5 articles report TTD results in PROs using QLQ-C30 [17, 19, 20, 22, 23]. The TTD was significantly longer with PD-1/PD-L1 inhibitors than with platinum-based chemotherapy in QoL, with an HR of 0.86 (95% *CI* = 0.76, 0.97; *P* = 0.013; Fig. 3A). There was no heterogeneity (*Q* = 3.28; *I*^2^ = 0.0%; *P* = 0.512). The *p-*value of Egger’s test is 0.057. Moreover, PD-1/PD-L1 inhibitors also demonstrated to delay TTD in physical function, pain, and role function on the QLQ-C30, with an HR of 0.79 (95% *CI* = 0.69, 0.90; *P* = 0.000; *I*^2^ = 67.0%; Fig. 3B), with an HR of 0.78 (95% *CI* = 0.69, 0.89; *P* = 0.000; *I*^2^ = 0.0%; Fig. 3C), with an HR of 0.84 (95% *CI* = 0.73, 0.96; *P* = 0.012; *I*^2^ = 74.3%; Fig. 3D), respectively. Sensitivity analysis of TTD was shown in Fig. 4 A–D.

### The difference of mean changes between groups

**Fig. 5:**
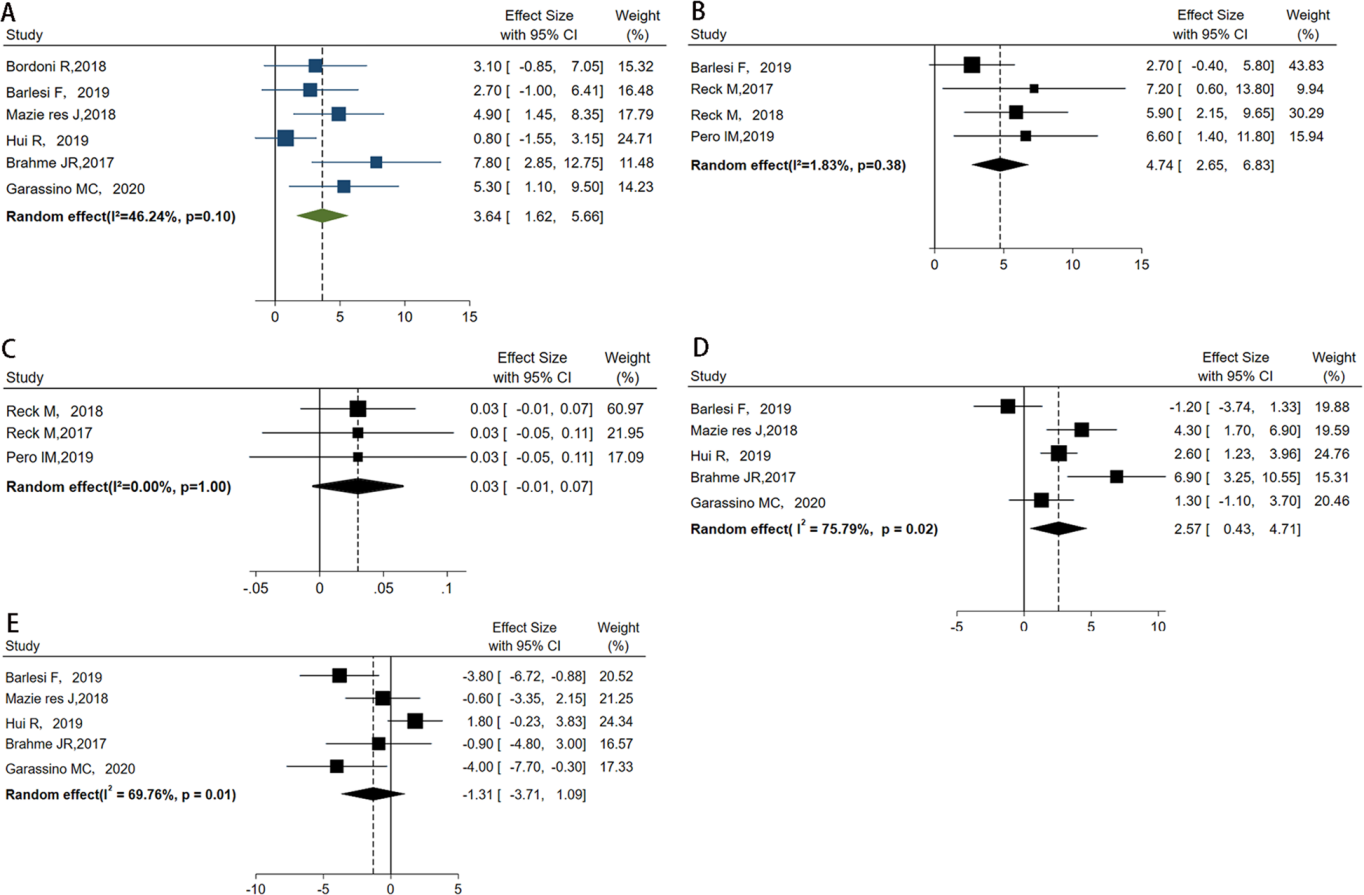
Forest plot of the difference in mean change from baseline to follow-up between groups for the Quality of Life Questionnaire Core 30 items (**A**), EQ-5D VAS (**B**), and EQ-5D utility index (**C**). **A** Random effect: *P* = 0.00; Egger’s test: *P* = 0.431; Begg’s test: *P* = 1.000. **B** Random effect: *P* = 0.00; Egger’s test: *P* = 0.128; Begg’s test: *P* = 0.296. **C** Random effect: *P* = 0.094; Begg’s test: *P* = 0.296. Forest plot of the difference in mean change from baseline to follow-up within groups: PD-1/PD-L1 inhibitors (**D**) and controls (**E**). **D** Random effect: *P* = 0.019; Egger’s test: *P* = 0.969; Begg’s test: *P* = 1.000. **E** Random effect: *P* = 0.284; Egger’s test: *P* = 0.141; Begg’s test: *P* = 0.462

The mean changes from baseline to follow-up in QoL between groups were reported with the QLQ-C30 in six trials [14, 16, 17, 19, 20, 22], the EQ-5D utility index in three trials [15, 18, 21], and the EQ-5D VAS in four trials [14, 17, 18, 21]. The difference in mean change between the PD-1/PD-L1 inhibitors group and the chemotherapy group in QLQ-C30 was 3.64 (95% *CI* = 1.62, 5.66; *P* = 0.00; Fig. 5A) favoring PD-1/PD-L1 inhibitors, with no heterogeneity (*Q* = 9.30; *I*^2^ = 46.24%; *P* = 0.10) and no publication bias (Begg’s test *P* = 1.000, Egger’s test *P* = 0.431). In addition, the same trend was also found for the EQ-5D VAS with a mean difference of 4.74 (95% *CI* = 2.65, 6.83; *P* = 0.00; Fig. 5B) and with no heterogeneity (*Q* = 3.06; *I*^2^ = 1.83%; *P* = 0.383). However, the pooled difference in mean change between PD-1/PD-L1 inhibitors and control groups in the EQ-5D utility index was 0.03 (95% *CI* = −0.01, 0.07; *P* = 0.094; *I*^2^ = 0.00%; Fig. 5C) with no statistically significant difference. Sensitivity analysis of the difference of mean changes between groups was shown in Fig. 4 E–G.

### The difference of mean changes within groups

There were eight trials accessible for the within-group analysis [14, 16, 19, 20, 22]. The pooled mean change from baseline to follow-up in PD-1/PD-L1 inhibitors in the QLQ-C30 was 2.57 (95% *CI* = 0.43, 4.71; *P* = 0.019, *I*^2^ = 75.79%) and in chemotherapy was −1.31 (95% *CI* = −3.71, 1.09; *P* = 0.284; *I*^2^ = 69.76%, Fig. 5 D–E), suggesting a change within the PD-1/PD-L1 inhibitors group. Statistically significant heterogeneity was observed in both studies (*Q* = 16.52, *I*^2^ = 75.79%, *P* = 0.002; *Q* = 13.23, *I*^2^ = 69.76%, *P* = 0.010). Sensitivity analysis of the difference of mean changes within groups was shown in Fig. 4 H–I.

### Subgroup analysis

**Fig. 6:**
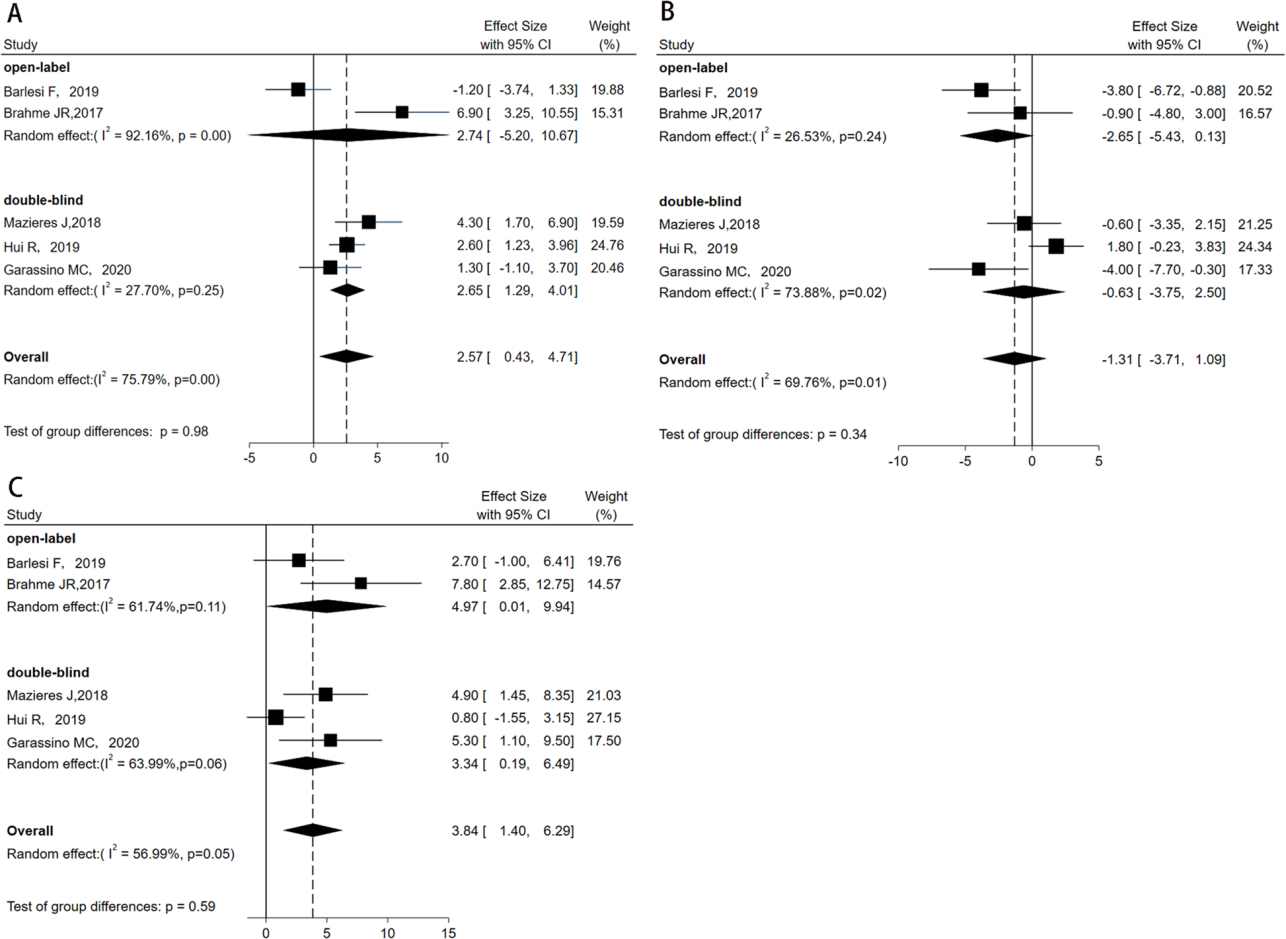
In global QLQ-C30, subgroup analysis (open-label trials versus double-blind trials) was performed on the mean difference within the PD-1/PD-L1 inhibitors group (**A**), within the chemotherapy group (**B**), and between the PD-1/PD-L1 inhibitors group and chemotherapy group (**C**) according to the masking

To further evaluate the impact of masking on QoL, we compared the effect of open-label trials versus double-blind trials in patients with NSCLC who underwent PD-1/PD-L1 inhibitors and chemotherapy in the QLQ-C30. The pooled mean change from baseline to follow-up in PD-1/PD-L1 inhibitors using open-label method was 2.74 (95% *CI* = −5.20, 10.67), with significant heterogeneity (*I*^2^ = 92.16%, *P* < 0.01). The pooled mean change of double-blind trials in PD-1/PD-L1 inhibitors group was 2.65 (95% *CI* = 1.29, 14.01; *P* = 0.25; *I*^2^ = 27.70%, Fig. 6A), suggesting low heterogeneity and beneficial change within the PD-1/PD-L1 inhibitors group [14, 16, 19, 20, 22]. The pooled mean change from baseline to follow-up in control groups using open-label method was −2.65 (95% *CI* = −5.43, 0.13), with low heterogeneity (*I*^2^ = 26.53%, *P* = 0.24). The pooled mean change of double-blind trials in control groups was −0.63 (95% *CI* = −3.75, 2.50, *P* = 0.02; *I*^2^ = 73.88%, Fig. 6B), suggesting high heterogeneity within the control group [14, 16, 19, 20, 22]. Whether open-label or double-blind trials, the mean changes from baseline to follow-up in QoL between groups both supported the PD-1/PD-L1 inhibitors group (Fig. 6C) [14, 16, 19, 20, 22].

## Discussion

This study compared the TTD and the difference of mean changes in PROs in patients with NSCLC receiving PD-1/PD-L1 inhibitors and chemotherapy. Regardless of QLQ-C30 or EQ-5D-3L, the results of the meta-analysis suggested that NSCLC patients with PD-1/PD-L1 inhibitors had a more favorable difference in mean change from baseline to follow-up compared to those with chemotherapy and had a significantly delayed clinical deterioration. The changes of QoL between groups showed that PD-1/PD-L1 inhibitors were more beneficial than chemotherapy; furthermore, there was a statistically significant favorable change among NSCLC treated with PD-1/PD-L1 inhibitors in the QLQ-C30 during the follow-up duration. EQ-5D-3L is the preference-based measure that could generate health utilities from results. By contrast, QLQ-C30 is the cancer-specific quality-of-life questionnaire, which focuses on non-preference-based patient outcomes and is usually applied in clinical trials. Therefore, different measures probably generate different results [27]. The subgroup analysis showed that no difference was observed between open-label and double-blind trials in patients treated with chemotherapy or PD-1/PD-L1 inhibitors. Furthermore, a preliminary analysis of PROs of identical oncology drugs found no overestimation of improvements in open-label trials compared to blinded trials [28]. Due to the small sample size, these results should be interpreted cautiously, and more data are needed for analysis. Recently, a meta-analysis demonstrated that ICIs were positively associated with higher levels of QoL and longer time to deterioration in cancer patients, which was consistent with our results, but the study incorporated a variety of cancer types and was not specific to PD-1/PD-L1 inhibitors, thus was not as homogenous as our study [29]. The adverse reactions often occur late in the course of therapy; the QLQ-C30 and EQ-5D-3L focus on the era of chemotherapy, which fails to include specific symptoms of ICIs, so account for ICI-treated patients may benefit more than chemotherapy-treated patients. Zaim R. et al. reported that nivolumab alleviated symptom burden and improved health-related quality of life of advanced NSCLC [30]. Nishijima T. F. et al. similarly found that patients treated with PD-1/PD-L1 inhibitors had higher QoL and fewer adverse effects than chemotherapy, yet there was no significant change in the mean difference in patients with PD-1/PD-L1 inhibitors, which contradicted our findings [31]. The reason may be associated with the different subjects, small sample size, and the failure to include specific symptom toxicities associated with ICIs in lung cancer [32].

To the best of our knowledge, our study is the first systematic review and meta-analysis examining the impact of PD-1/PD-L1 inhibitors and standard chemotherapy on QoL in patients with NSCLC. However, there are some limitations to our study. Firstly, these data were extracted from published articles and were not original. Secondly, fewer articles were eligible for inclusion, and subgroup analysis of heterogeneous studies was difficult to conduct with some publication bias. Thirdly, as QoL questionnaires require patients to complete at a point time, the patients are vulnerable to losing follow-up due to the disease progression. Fourthly, it is difficult to directly compare different trials at different time points, and other functions and symptoms of PROs cannot be studied due to a lack of data and heterogeneity. Lastly, despite questionnaires reporting quality of life are currently considered to be of high quality, there are currently no international guidelines for statistical analysis of QOL in cancer patients using ICIs, and there is still a lack of confirmation of research hypotheses and questionnaires in cancer patients treated with ICIs in methodological aspects [33]. ECOG PS ≥ 2 was almost excluded from clinical trials, and future studies should be focused on older and weak patients [34, 35].

## Conclusion

Our meta-analysis reported that patients with NSCLC treated with PD-1/PD-L1 inhibitors had higher QOL and fewer adverse symptoms than those with standard chemotherapy, indicating that early social, psychological, and spiritual support can improve the quality of life. The shift from traditional therapy to immunotherapy in cancer treatment will take the consideration of the patient-centered quality-of-life assessment into account, which can have a positive impact on the development of oncological care in the future. It has reference value for clinicians in the real world, and more studies will be needed to validate it.

## Data Availability

All data in this manuscript are available for readers.
